# Silencing *BMP-2* expression inhibits A549 and H460 cell proliferation and migration

**DOI:** 10.1186/1746-1596-9-123

**Published:** 2014-06-19

**Authors:** Heying Chu, Hailan Luo, Huaqi Wang, Xiaonan Chen, Ping Li, Yong Bai, Furui Zhang, Ruirui Cheng, Shanshan Chen, Yuanyuan Wang, Guoqiang Zhao, Guojun Zhang

**Affiliations:** 1 Department of Respiratory Medicine, the First Affiliated Hospital of Zhengzhou University, Zhengzhou 450052, China; 2 Department of Medical Biology, Luohe Medical College, Luohe, Henan 462002, China; 3 College of Basic Medical Sciences, Zhengzhou University, No.100 Kexue Road, Zhengzhou 450001, China

**Keywords:** Non-small cell lung cancer (NSCLC), BMP-2, siRNA, Proliferation, Migration

## Abstract

**Abstract:**

**Background**

Bone morphogenetic protein 2 (BMP-2) is a member of the TGF-β superfamily that is closely correlated with many malignancies, particularly lung cancer. However, the effects of silenced *BMP-2* on lung cancer cell proliferation and migration are not clear.

**Methods**

Using quantitative real-time RT-PCR, *BMP-2* mRNA expression was detected in 61 non-small cell lung cancer (NSCLC) samples. Survival curves were generated using follow-up data. Relationships between clinical or pathological characteristics and prognosis were analyzed. Cell viability assays and transwell migration assays were used to evaluate the effects of *BMP-2* silencing on cell proliferation and migration of A549 and H460 cells.

**Results**

*BMP-2* mRNA expression was higher in NSCLC tissues compared to matched adjacent normal tissues (*P* < 0.01). High *BMP-2* expression levels were significantly associated with the occurrence of lymph node metastases and tumor stage (*P* < 0.05). There were significant differences in survival curves between groups with metastatic lymph nodes and non-metastatic lymph nodes, as well as between groups with low *BMP-2* expression and groups with high *BMP-2* expression. In addition, we observed decreased proliferation and migration rates of the NSCLC-derived cell lines A549 and H460 that were transfected with *siBMP-2* (*P* < 0.05).

**Conclusion**

*BMP-2* mRNA is overexpressed in NSCLC samples and is a risk factor for survival in patients with NSCLC. *BMP-2* silencing can significantly inhibit A549 and H460 cell proliferation and migration.

**Virtual slides:**

The virtual slide(s) for this article can be found here: http://www.diagnosticpathology.diagnomx.eu/vs/4263254471298866

## Background

Lung cancer is one of the most common causes of cancer-related deaths, and its incidence is increasing [1-3]. Approximately 80% of diagnosed lung cancer cases are non-small-cell lung cancer (NSCLC) [4,5]. Despite improvements in diagnosis and treatment, the long-term survival rate has only marginally improved.

Bone morphogenetic protein 2 (BMP-2) was originally identified as an osteoinductive cytokine, and was subsequently reported to have an important role in cell migration, proliferation, and differentiation [6,7]. Serum BMP-2 levels from NSCLC samples were higher compared to healthy controls, and positively correlated with poor prognosis, stage, and metastatic burden [8,9]. BMP-2 protein expression in human NSCLC is higher than in normal lung tissue, and recombinant BMP-2 promotes cell migration and invasiveness [10]. Additionally, tumor growth was promoted in nude mice that were injected with A549 cells that were transfected with recombinant BMP-2 [11]. Finally, BMP-2 is overexpressed in the majority of lung carcinomas and stimulates the growth and progression of lung tumors [12]. However, the effects of silencing *BMP-2* on lung cancer cell proliferation and migration were not clear.

In this study, we used siRNA to silence *BMP-2* to observe the effect on proliferation and migration of the lung cancer cell lines A549 and H460. Moreover, we analyzed the correlation between *BMP-2* mRNA expression and the clinicopathological characteristics of 61 patients with NSCLC.

## Methods

### Clinical sample collection

In this study, 61 patients with NSCLC from the First Affiliated Hospital of Zhengzhou University were enrolled between 2003 and 2008. Patients who had recurrent or primary NSCLC but received chemoradiotherapy before surgery were excluded. Of the 61 patients, 32 were female and 29 were male. Twenty-eight cases had lymph node metastases, whereas 33 cases did not. We obtained paired NSCLC and adjacent non-tumor lung tissues (located more than 5 cm away from the tumors) from 61 patients who underwent primary surgical resection of NSCLC. All patients provided informed consent. Both tumor and non-tumor samples were confirmed as such by pathological examinations. These samples were snap-frozen in liquid nitrogen after resection. The Human Research Ethics Committee of Zhengzhou University approved this study (Table 1).

**Table 1:** **Clinicopathological characteristics and *BMP-2 *** **mRNA expression in NSCLC patients (N = 61)**

**Parameter**	**n**	**Expression level**	***P*-value**
Sex			0.415
Male	29	0.8383 ± 0.13541	
Female	32	0.8692 ± 0.15649	
Age (years)			0.312
≥60	33	0.8369 ± 0.12853	
<60	28	0.8753 ± 0.16512	
Differentiation			
Well	19	0.8704 ± 0.15222	0.573
Moderate-poor	42	0.8473 ± 0.14509	
Tumor stage			0.014*
T1	18	0.8260 ± 0.13904	
T2	31	0.8290 ± 0.14797	
T3–4	12	0.9632 ± 0.1066	
TNM stage			0.341
I	26	0.8356 ± 0.13043	
II	22	0.8458 ± 0.17847	
III	13	0.9071 ± 0.11028	
Nodal status			0.026*
Positive	28	0.8994 ± 0.15196	
Negative	33	0.8164 ± 0.13218	

*Indicates statistical significance (*P* < 0.05).

### Cell lines and cell culture

The human lung cancer cell lines A549 and H460 were maintained in DMEM supplemented with 10% fetal bovine serum (FBS; Gibco), 100 units/mL penicillin, and 100 μg/mL streptomycin in a humidified incubator of 5% CO_2_ at 37°C.

### Main materials

DMEM (coring, USA); Cell Counting Kit-8 (Dojindo Laboratories, Japan); hematoxylin (sigma, USA); quantitative real time PCR assay kit(SYBR Premix Ex Taq)(TaKaRa, Japan); Trizol (Invitrogen, USA); AMV reverse transcriptase (Promega, USA); the first antibody of BMP-2, β-actin (Santa Cruz Corp, USA); the goat anti-rabbit horseradish peroxidase-labeled secondary antibody (Bio-Rad, USA); chemiluminescence substrate kit (Amersham, USA); NSCLC-derived cell lines A549 and H460 were obtained from (ATCC, USA).

### RNA oligo-ribonucleotides and cell transfection

si*BMP-2* and negative control (NC) sequences were as follows: si*BMP-2*: sense: 5′-AATAGCAGTTTCCATCACCGA-3′; anti-sense: 3′-TTATCGTCAAAGGTAGTGGCT-5′; negative control: sense: 5′-ATACTATTCCGAGCGACATAC-3′; anti-sense: 3′-TA TGATAAGGCTCGCTGTATG-5′. Sequences were chemically synthesized by Shanghai GenePharma Co., Ltd. A549 and H460 cells were seeded into six-well plates (2 × 10^5^ cells/well). Transfection was performed by electroporation. Three groups were generated for the ensuing experiments: non-transfected group (blank control), siRNA negative control-transfected group (NC), and si*BMP-2* transfected group (siRNA *BMP-2*). Cells were harvested for experiments 24–48 h post-transfection.

### Cell counting kit-8 assays

We used the Cell Counting Kit-8 (Dojindo Laboratories, Japan) according to the manufacturer’s instructions to determine cell viability. Briefly, cells were seeded at a density of 2 × 10^3^ cells/well in 96-well plates (in three replicate wells) and treated daily for 4 consecutive days with 10 μl/well of Cell Counting Kit-8 solution. Optical density was measured at 450 nm to estimate the number of viable cells.

### Transwell migration assays

We assayed the migration ability of cells using 6.5 mm diameter transwell chambers with 8 μm membranes (Corning, USA). Twenty-four hours post-transfection, A549 and H460 cells were seeded in the upper chambers, and the bottom wells were coated with 1 mg/ml matrigel for migration assays. Media containing 10% FBS were added to the bottom chambers. After 24 h at 37°C in a 5% CO_2_ humidified atmosphere, cells in the upper chamber were carefully scraped off using a cotton swab, and cells that had migrated to the basal side of the membrane were fixed in methanol, stained with hematoxylin, and counted. Each test was performed in triplicate.

### RNA extraction and quantitative real-time RT-PCR

We isolated total RNA from tissue samples and transfected cells using Trizol, and cDNAs were generated using AMV reverse transcriptase. BMP-2 primers were designed using Oligo 7.0 software according to the *BMP-2* mRNA sequence (NM_001200). Sequences were as follows: *BMP-2* forward 5′-ATAGCAGTTTCCATCACCGAA-3′, reverse 5′-ACTTCCACCACGAAT CCAT-3′; β*-actin* forward 5′-AAAGACCTGTACGCCAACACA-3′, reverse 5′-CGATCCACACGGAGTACTTGC-3′. Primers were synthesized by Sangon Biotech (Shanghai) Co., Ltd. Real-time RT-PCR was performed in triplicate on the ABI 7500 Fast Real-time PCR system. Cycling parameters were 35 denaturation cycles of 95°C for 15 s, annealing at 55°C for 30 s, and elongation at 72°C for 30 s. Gene expression was quantified using the comparative CT method, normalizing CT values to the housekeeping gene β*-actin*. After amplification, melting curve analyses were performed to ensure the specificity of the products.

### Western blotting

Total protein was extracted from transfected cells, and protein concentrations were measured using Bradford assays. Protein lysates (25 μg) were subjected to SDS-PAGE. Electrophoresed proteins were transferred to nitrocellulose membranes (Whatman, USA). After blocking in 5% non-fat milk, membranes were washed at room temperature and incubated with the following primary antibodies: BMP-2 (1:1000; Santa Cruz Biotechnology, USA) and β-actin (1:1000; Santa Cruz Biotechnology). Following extensive washing, membranes were incubated for 1 h with the goat anti-rabbit horseradish peroxidase-labeled secondary antibody (1:3000; Bio-Rad, USA). An enhanced chemiluminescence substrate kit (Amersham, USA) was used to detect signals with autoradiography film (Amersham).

### Statistical analyses

Statistical analyses were performed using SPSS 17.0 software (SPSS Inc., USA). Data are expressed as the mean ± standard deviation (SD). Student’s t-tests were used to compare the mean between two samples. Logistic analyses were used in the correlation of lymph node metastasis with *BMP-2* mRNA expression. Follow-up data were analyzed using the Kaplan–Meier method and log-rank tests. P-values less than 0.05 were considered statistically significant.

## Results

### *BMP-2* mRNA is overexpressed in NSCLC samples

To explore the relationship between *BMP-2* mRNA expression and NSCLC clinicopathological characteristics, we retrospectively analyzed 61 NSCLC patients. We evaluated the expression of *BMP-2* mRNA using quantitative real time RT-PCR, and explored the relationship between *BMP-2* mRNA levels and TNM stage, node status, gender, age, differentiation, and tumor stage. Our data analyses showed that the relative expression levels of *BMP-2* mRNA in cancer tissues (61 samples, 0.8545 ± 0.14650) were significantly higher compared to matched adjacent normal tissues (61 samples, 0.1386 ± 0.0285) (*P* = 0.000). In addition, there were higher *BMP-2* mRNA levels in NSCLC samples with lymph node metastasis (28 samples, 0.8994 ± 0.15196) compared to NSCLC samples without lymph node metastasis (33 samples, 0.8164 ± 0.13218) (*P* = 0.026), as well as in tumor stage T3–4 samples (12 samples, 0.9632 ± 0.1066) compared to tumor stage T1 samples (18 samples, 0.8260 ± 0.13904) or T2 samples (31 samples, 0.8290 ± 0.14797) (*P* = 0.014) (Table 1). These results indicate that *BMP-2* expression positively correlates with tumor stage and lymph node metastasis in NSCLC patients.

### *BMP-2* mRNA expression level is a risk factor for survival in patients with NSCLC

**Figure 1:**
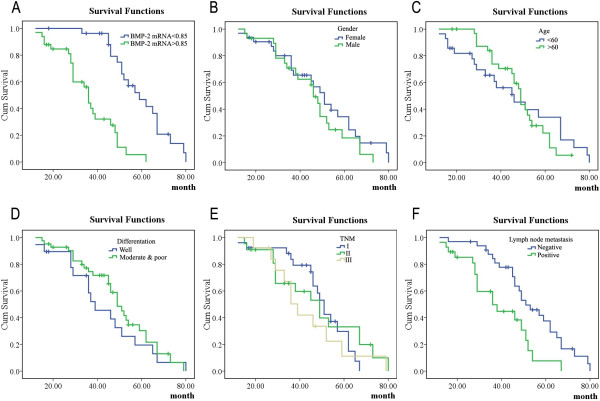
**Kaplan–Meier survival analyses. A**: Schematic representation of survival curves from groups expressing different *BMP-2* mRNA levels (*P* = 0.000). **B**: Schematic representation of survival curves of different genders in patients with NSCLC (*P* = 0.309). **C**: Schematic representation of survival curves of different ages in patients with NSCLC (*P* = 0.800). **D**: Schematic representation of survival curves of tumor differentiations in patients with NSCLC (*P* = 0.355). **E**: Schematic representation of survival curves of different TNM stages in patients with NSCLC (*P* = 0.518). **F**: Schematic representation of survival curves from the lymph node metastatic group and non-metastatic group in patients with NSCLC (*P* = 0.004).

Survival curves were generated using the Kaplan–Meier method. Log-rank tests indicated that there were significant differences in survival curves from groups expressing different *BMP-2* mRNA levels (χ^2^ = 27.108, *P* = 0.000, Figure 1A), as well as between the lymph node metastatic group and non-metastatic group (χ^2^ = 8.372, *P* = 0.004, Figure 1F). However, there were no significant differences between male and female patients (χ^2^ = 1.037, *P* = 0.309, Figure 1B), patients ≥60 years versus patients <60 years old (χ^2^ = 0.064, *P* = 0.8, Figure 1C), or patients with well differentiated tumors versus those with moderately to poorly differentiated tumors (χ^2^ = 0.857, *P* = 0.355, Figure 1D). Similarly, there were no significant differences among the TNM stage I, II, and III groups (χ^2^ = 1.317, *P* = 0.518, Figure 1E). Logistic regression analysis indicated that the relative expression level of *BMP-2* mRNA was a risk factor for lymph node metastasis in patients with NSCLC.

### Effects of siRNA-*BMP-2* on cell proliferation

**Figure 2:**
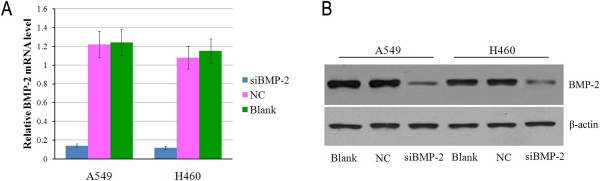
**Effects of si*BMP-2 *on *BMP-2 *mRNA and protein expression in A549 and H460 cells.** A549 and H460 cells were transfected with si*BMP-2* or NC. **A**: *BMP-2* mRNA expression in cells transfected with si*BMP-2* decreased significantly compared to untransfected cells. **B**: BMP-2 protein expression in si*BMP-2-*transfected cells was lower compared to control groups (NC and blank).

**Figure 3:**
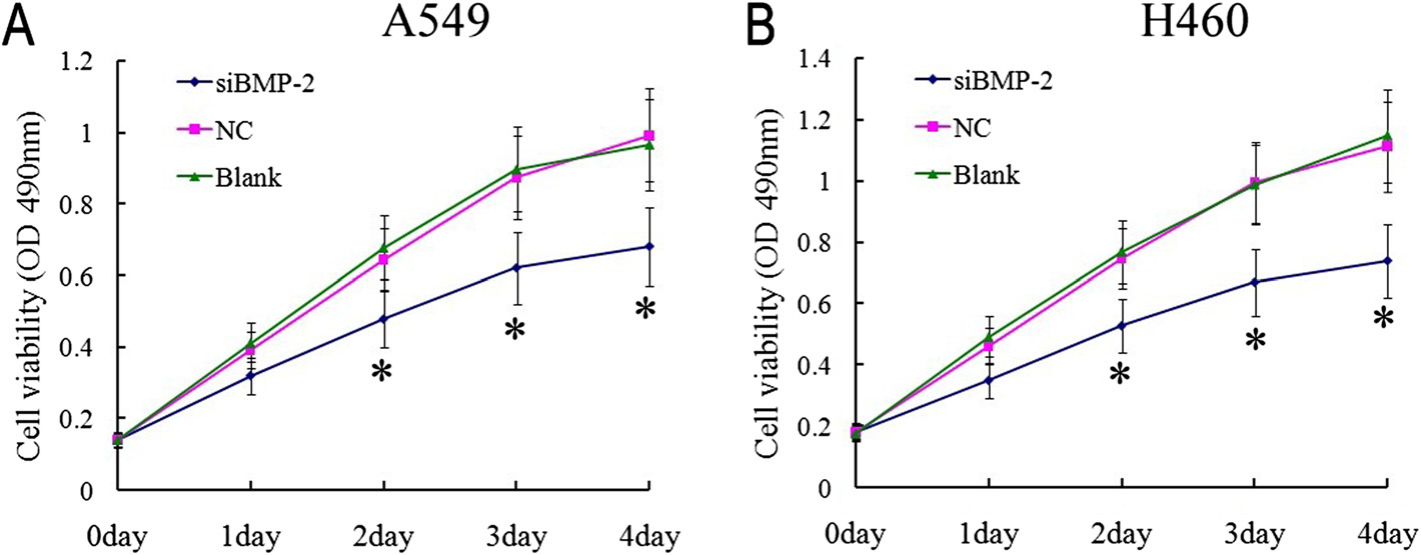
**Cell proliferation was assessed using CCK-8 assays.** A549 and H460 cells were transfected with si*BMP-2* or NC. Data are presented as the mean of four experiments. Compared with control groups (blank and NC), *BMP-2* silencing significantly decreases proliferation of the lung carcinoma cell lines A549 **(A)** and H460 **(B)** after 2, 3, and 4 days (*P* < 0.05).

RT-PCR results showed that the mRNA expression of *BMP-2* in A549 and H460 cells transfected with si*BMP-2* was decreased compared to cells transfected with NC and blank control (*P* < 0.05, Figure 2A). Further, we examined BMP-2 protein expression using western blot analyses and found the results were consistent with the mRNA data (*P* < 0.05, Figure 2B). These results demonstrate the silencing effect of *BMP-2* siRNA. According to the CCK-8 assay results, the absorbance of A549 and H460 cells transfected with si*BMP-2* was significantly reduced after 2, 3, and 4 days compared with the control cells (NC and blank) (*P* < 0.05, Figure 3). These results demonstrate that silencing *BMP-2* significantly inhibits A549 and H460 cell proliferation.

### Effects of si*BMP-2* expression on cell migration

**Figure 4:**
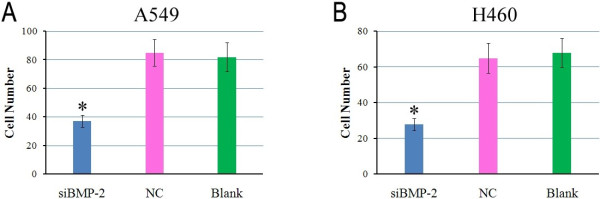
**si*BMP-2 *inhibits cells migration.** A549 and H460 cells were transfected with si*BMP-2* or NC. Histograms show significantly fewer migrating cells upon transfection with siBMP-2 compared to the control groups (NC and blank) in A549 **(A)** and H460 **(B)** cells.

In order to explore the effect of *BMP-2* siRNA on cell migration, we performed transwell migration assays. We found that the number of si*BMP-2*-transfected A549 or H460 cells that traveled through the micropore membrane was lower compared to the control groups (NC and blank) (*P* < 0.05; Figure 4A, B). This result indicates that downregulation of *BMP-2* inhibits the migratory ability of A549 and H460 cells *in vitro.*

## Discussion

Bone morphogenetic proteins (BMPs) are members of the TGF-β superfamily and are aberrantly expressed in many types of carcinoma cells, including prostate, lung, breast, gastric, and ovarian [13-16]. BMP-2 is known to stimulate proliferation, differentiation, and migration during embryonic development [6,17-21]. BMP-2 abrogated the fibrogenic function of TGF-β in pancreatic stellate cells via the Smad1 signaling pathway [22]. Moreover, high concentrations of BMP-2 strongly enhanced gastric cancer cell motility and invasiveness [23]. BMP-2 upregulation caused epithelial dysfunction and hyperpermeability [24], and enhanced the neovascularization of developing lung tumors. BMP-2 is aberrantly expressed in approximately 98% of lung carcinomas [25]. BMP-2 is highly overexpressed in human NCSLC compared with normal lung tissue and benign lung tumors, and high BMP-2 levels enhanced tumor cell migration and invasion, thereby promoting tumor growth [10,11,26,27]. Thus, these data indicate that BMP-2 has important biological activity in lung carcinomas and a potential marker of lung carcinomas. Up to now, several lung carcinomas potential markers had reported, such as Tiam1, MAT3, DNA methylome [28-30].

In this study, we observed that the mRNA expression of BMP-2 in tumor tissue was significantly higher than in matched adjacent normal tissues (*P* < 0.01). Furthermore, we found that B*MP-2* expression was related to lymph node metastasis, tumor stage, and survival time. These results suggest that BMP-2 may play a role in tumor metastasis.

High levels of *BMP-2* promote tumorigenesis. However, downregulation of *BMP-2* reduced tumor growth. For example, inhibition of *BMP-2* activity using either recombinant Noggin or a BMP-2 antibody caused a reduction in lung tumor growth [10]. Blocking BMP signaling with the inhibitor DMH1 reduced lung cell proliferation, promoted cell death, and decreased cell migration and invasion in NSCLC cells [31]. *BMP-2* knockdown by adenovirus inhibited growth and invasion of human lung adenocarcinoma cells by blocking PI3K/AKT signaling [32]. In this study, we suppressed *BMP-2* activity by siRNA. These data show that suppressing BMP-2 expression significantly inhibited lung tumor cell proliferation and migration (Figures 3 and 4). This outcome is in accordance with previous studies and further confirms the biological function of BMP-2 in lung cancer.

Previous studies of BMP-2 have focused on the expression of BMP-2 in tumor tissues and its function in tumor cell proliferation, invasion, and migration. However, for the first time, we investigated and assessed the relationship between *BMP-2* expression and clinicopathological characteristics. Our analyses found significant correlations between *BMP-2* expression and lymph node metastasis, TNM stage, tumor stage, and survival time (Figure 1). Transwell migration assays also showed that the number of si*BMP-2*-transfected cells that migrated decreased. This result suggests that *BMP-2* expression is closely related to lung tumor metastasis.

## Conclusion

In summary, our data show that *BMP-2* silencing in the lung cancer cell lines A549 and H460 suppressed their proliferation and migration, thereby suggesting that BMP-2 might be a novel therapeutic strategy for human NSCLC.

## Abbreviations

NSCLC: Non-small-cell lung cancers; BMP-2: Bone morphogenetic protein 2.

## Competing interests

The authors declare that they have no competing interests.

## Authors’ contributions

GJZ, HYC and GQZ: conceived of the study, and participated in its design and coordination and helped to draft the manuscript. HYC, HLL, YB, FRZ, RRC, SSC and YYW: collected the samples. HYC, HLL, HQW, XNC and PL: carried out part of experiments and wrote the manuscript. HYC, YYW, GJZ and GQZ performed the statistical analysis. All authors read and approved the final manuscript.
